# Potential mediators of the link between wealth index and anthropometric indices of under-five children in Ethiopia

**DOI:** 10.3389/fpubh.2022.981484

**Published:** 2022-10-13

**Authors:** Lijalem Melie Tesfaw, Ashenafi Abate Woya

**Affiliations:** Department of Statistics, Bahir Dar University, Bahir Dar, Ethiopia

**Keywords:** anthropometric indices, Ethiopia, mediation analysis, wealth index, structural equation (SEM)

## Abstract

**Background:**

Malnutrition of under-five children has adverse effects on child's health and development, such as growth failure and muscle wasting. The household wealth index has a direct contribution to child malnutrition despite little attention being given to its indirect effect through other factors. This study aimed to identify the potential mediators of the link between wealth index and malnutrition.

**Methods:**

In this study, a cross-sectional study design was implemented based on the data obtained from the 2019 Ethiopia Mini Demographic and Health Survey (EMDHS), which consists of a total of 3,918 under-five children. Mediation analysis of structural equation (SEM) was implemented to determine and estimate the effects of potential mediators of the link between wealth index and under-five children malnutrition.

**Results:**

Among 3,918 under-five children involved in this study, 51.4% were male children and 20.2% were from urban areas. The place of delivery of the majority of mothers (56.1%) was at home, and the majority of children were from the poorest household (36.4%). The estimated effects of height-for-age of female children were higher (1.31:95% CI = 0.45, 0.200), indicating that female children had a lower likelihood of stunting than males. As compared to children in an urban area, children from rural areas were more likely to be stunted (−0.269; 95%CI = −0.388, −0.135). The estimated total indirect effects of wealth index on stunting and underweight was 0.69 (95% = 0.045, 0.094) and 0.036 (95% = 0.013, 0.054), respectively, and significant (*p*-value of <0.05).

**Conclusion:**

Environmental, maternal, biological, and behavioral factors were the potential mediators of the link between the wealth index and the anthropometric indices. Besides, the total effects of the household wealth index had a significant effect on stunting, underweight, and wasting in which children from a household with low economic status were more likely to be malnourished.

## Background

Malnutrition of under-five children has adverse effects on child's health and development, such as growth failure and muscle wasting (1). Malnutrition in children is mainly responsible for reduced immunity for the prevention of diseases. Due to this fact, reducing malnutrition is the most important constituent of the Sustainable Development Goals (SDGs) (2). Malnutrition enables to aggravate the risk of death, morbidity, and infection and reduces the mental, physical and psychological development of children during their early ages (2, 3). However, the common type of malnutrition that occurred in developing countries, such as sub-Saharan Africa, including Ethiopia is undernutrition. In developing countries like Ethiopia, the prevalence of undernutrition of under-five children remains a series problem (3, 4).

Though malnutrition, in particular, undernutrition (stunting, underweight, and wasting), is a universal health problem, it is common in low- and middle-income countries. According to a 2015 World Health Organization report, globally, 26, 16, and 8% of children under five were stunted, underweight, and wasting, respectively (5, 6). The highest proportion of undernourished under-five children (more than 90%) lives in Africa and Asia (5).

Maternal education, age, residence, religion, body mass index, access to health care, and economic status are among the most prevalent determinants of malnutrition (2–4). Mothers' body mass index was also significantly associated with a child's undernutrition in which lower BMI was linked with a child's underweight (7). Among several determinants of malnutrition, household economic status shares the highest contribution that influences the dietary intake of children and mothers (8). A lower household wealth index leads to inadequate food supplies, use of health services, availability of improved water sources, and sanitation facilities (3, 8).

The conventional and well-known indicators of malnutrition are stunting, wasting, and being underweight (4). These indicators of child malnutrition are comprehensively also called composite indices of anthropometric failure (CIAF). In all measures of child anthropometric failure, the burden of child undernutrition remains high. Child nutritional status depends on the level of household income. It is understood that household income interacts with household food insecurity, inadequate care, and an unhealthy environment that induce morbidity and mortality in children (8).

Numerous biological, behavioral, demographic, and social-economic underlying determinants contribute to malnutrition (8, 9). Over half of childhood deaths in developing countries result from undernutrition (9). Undernourished children are easily vulnerable to increased risk of communicable and non-communicable diseases. The primary important determinant of children's malnourished status is dietary intake and health status. The dietary intake of children directly depends on the properties or wealth index or economic status of the household/parents/guardian (3, 9). The lower economic status of a household is associated with a higher likelihood of undernutrition.

Numerous studies (3, 10, 11) demonstrated factors, mainly wealth index, contributing to malnutrition among under-five children and have been stable to date in developing countries in particular. However, the contribution of the wealth index to malnutrition may be mediated by other socioeconomic, demographic, behavioral, and biological factors despite little attention being given. Taking into account these mediators is essential for decision, policy, and plan makers to improve the adverse effects of malnutrition (12). As a result, implementing mediation analysis of structural equation modeling in this study was more advantageous than other studies that implemented traditional methods, such as regression analysis, which are limited to detecting multi-directional linkage between variables beyond simple correlation (13–15). Sometimes, despite families having good economic status, children within a family are malnourished. These might be because of other mediator characteristics of the household, such as environmental, biological, maternal, and behavioral factors. For instance, most children in rural areas are malnourished as compared to children in the urban area as the family in rural areas have poor management habits despite they have adequate resources (3).

A study in Uganda (10) also indicates that nutritional deficiency links with household income. Thus, studies that determine factors that enable to explain through which economic status and undernutrition are related to each other are very limited. In several studies (8, 16, 17), thus far, direct effects of wealth index on undernutrition were estimated and it is perceived that wealth index is an important determinant. However, to the authors' knowledge, there are no study findings that show the indirect effects of wealth index through other biological, behavioral, demographic, and social-economic factors. Therefore, this study aimed to identify the potential mediators of the link between wealth index and malnutrition. This enables us to estimate the total effect (direct and indirect) of the wealth index on malnutrition. As a result, in effect analysis, it is often necessary to determine and separate the pathways that link wealth index to an outcome of interest stunting, underweighting, and wasting for better and flexible policy-making as well as other stakeholders to reduce the adverse effects on child health due to malnutrition.

## Methods

### Data source

In this study, a total of 3,918 under-five children were involved. The data for this study was obtained from the 2019 Ethiopia Mini Demographic and Health Survey (EMDHS). Providing up-to-date estimates of key demographic and health indicators by collecting high-quality data for maternal and child health are the primary objectives of EDHS among its multidisciplinary objectives (13). The sampling frame used for the 2019 EMDHS is a frame of all census enumeration areas (EAs) created for the 2019 Ethiopia Population and Housing Census (EPHC) and conducted by the Central Statistical Agency (CSA). The 2019 EDHS sample was stratified and selected in two stages. In the first stage, a total of 305 EAs were selected with probability proportional to EA size and with independent selection in each sampling stratum. A household listing operation was carried out in all selected EAs from January through April 2019. The resulting lists of households served as a sampling frame for the selection of households in the second stage. In the second stage of selection, a fixed number of 30 households per cluster were selected with an equal probability of systematic selection from the newly created household listing, and maternal as well as children characteristics were obtained *via* interview and questionnaire (13).

### Inclusion/exclusion criteria

The inclusion criteria were aged below 5 years and completed relevant forms about the personal information and clinical signs. Hence, children who had not completed all relevant information or aged ≥5 years were excluded.

### Variables

**Figure 1:**
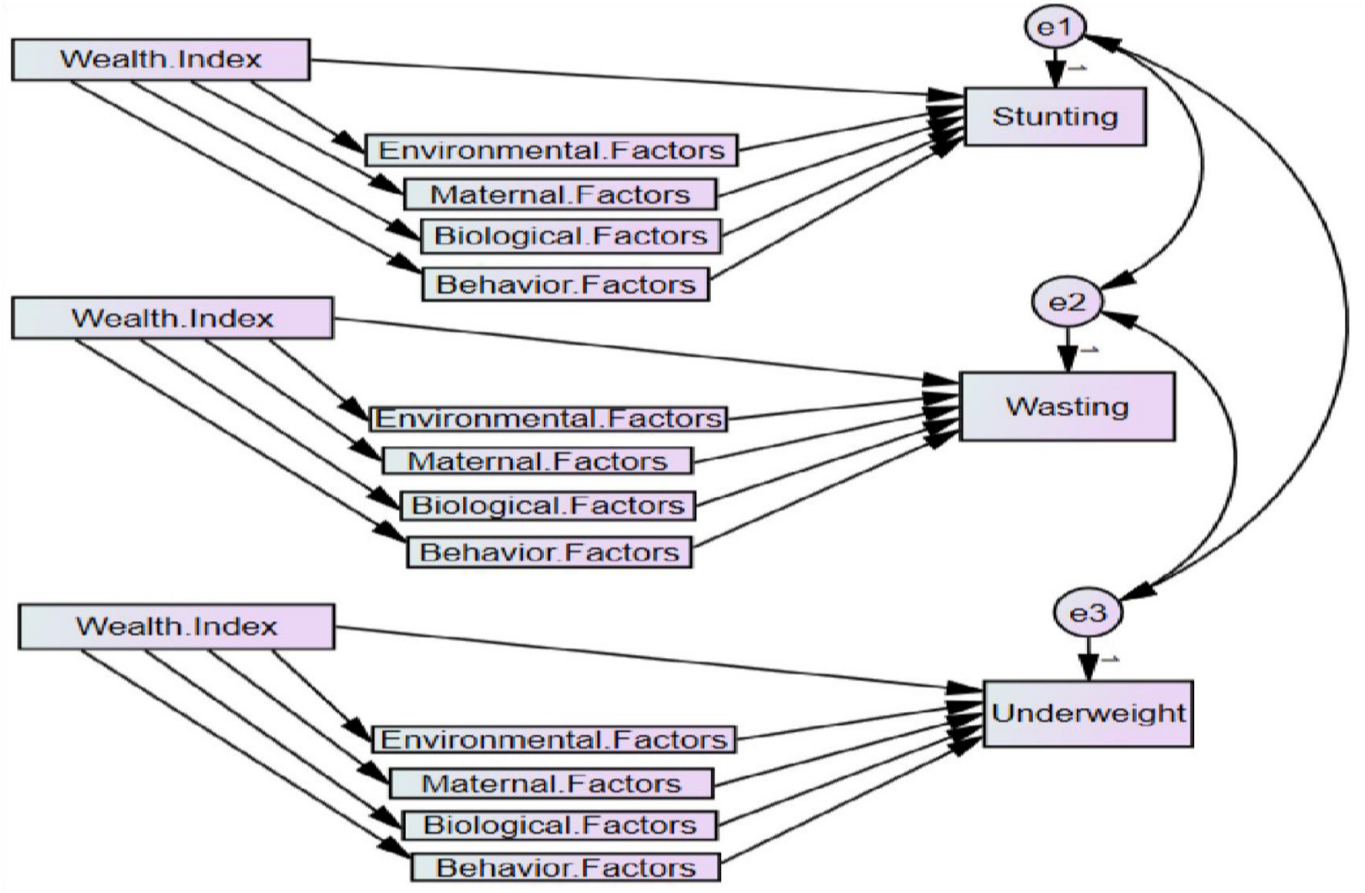
Hypothetical framework of mediators of the link between wealth index and anthropometric indices stunting, underweight, and wasting.

The dependent (anthropometric indices) and independent (mediators and wealth index) variables considered in this study are presented in Table 1. The mediators or independent variables were driven by a previous study (3, 8). Comprehensively, based on information obtained from studies (4, 8, 16), the theoretical framework for the study is presented in Figure 1. The environmental, maternal, biological, and behavioral factors are hypothetical mediators of the link between the wealth index and the anthropometric indices, such as stunting, wasting, and underweight, which are presented in Figure 1. The environmental factors: the residence and wealth indices; maternal factors: mothers' education level and mothers' age at first birth; biological factors: sex of the child, child's age, and birth interval; and behavior factors: place of delivery, contraceptive use, breastfeed, and family size. The single and double arrow of the path diagram in Figure 1 indicates the causal effect and correlation between variables, respectively.

**Table 1:** Independent variables description, frequency distribution, and summary statistics.

**Variables**	**Categories (codes)**	***n* (%)**	**Percent**
Sex of child	Male (1)	2,012	51.4
	Female (2)	1,906	48.6
Residence	Urban (1)	791	20.2
	Rural (2)	3,127	79.8
Place of delivery	Home (1)	2,197	56.1
	Health sector (2)	1,721	43.9
Breastfeed	No (0)	2,634	67.2
	Yes (1)	1,284	32.8
Contraceptive use	No (0)	1,217	31.1
	Yes (1)	2,701	68.9
Wealth index	Poorest (1)	1,426	36.4
	Poorer (2)	717	18.3
	Middle (3)	564	14.4
	Rich (4)	493	12.6
	Richest (5)	718	18.3
	**Mean (SD)**	**Minimum**	**Maximum**
Child age (in months)	29.54 (16.94)	1	59
Birth interval (in years)	3.03 (2.03)	1	14
Mothers age at first birth	18.08 (4.01)	12	44
Mother's education level (in years)	2.40 (3.969)	0	23
Family size	6.58 (2.19)	2	24
Stunting (HAZ)	−1.47 (1.58)	−6	5
Underweight (WAZ)	−1.20 (1.27)	−6	4
Wasting (WHZ)	−0.49 (1.17)	−5	5

**Figure 2:**
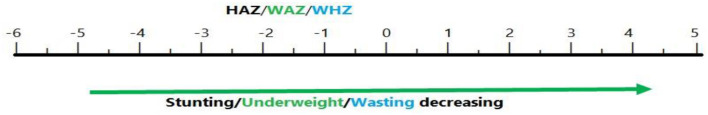
Values of HAZ/WAZ/WHZ and patterns of stunting/underweight/wasting.

The anthropometric indices stunting, underweight, and wasting are the standardized height-forage (HAZ), weight-for-age (WAZ), and weight-for-height (WHZ), respectively (18). Commonly, according to WHO standards, a child is stunted, underweight, and wasting if HAZ, WAZ, and WHZ are <-2, respectively. On the other hand, this indicates higher values of HAZ, WAZ, and WHZ refer to lower values of stunting, underweight, and wasting, respectively, see Figure 2. The minimum and maximum standardized score of HAZ, WAZ, and WHZ was −6 and 5, respectively (see Table 1 and Figure 2).

### Statistical analysis

#### Mediation analysis

Traditional multivariate modeling (linear regression, ANOVA, Poisson regression, logistic regression, and proportional hazard modeling) is useful for examining direct relationships between independent and dependent variables (14). However, real life may not be so parsimonious, and relationships between various variables may be much more complex, i.e., independent variables may have indirect effects on the dependent variable beyond their direct effect (14, 15). Such types of relationships could not be easily modeled with standard regression techniques. Structural equation modeling (SEM) readily allows for the exploration of such complex interrelationships (15, 19). SEM commonly includes mediation/path analysis, exploratory analysis, and confirmatory analysis (10, 12). In this study, mediation analysis of SEM was used (13).

In mediation, an intermediate variable or mediator helps to explain how or why an independent variable influences an outcome. This is used to identify and study the mechanisms by which an exposure achieves its effect on the outcome of interest (15). In the case of mediation analysis, quantitative estimates of the coefficients, including direct, indirect (*via* mediator), and total effects based on observed correlations among variables can be generated (12). The mediation analysis consists of comparing two regression models, one with and one without conditioning on the mediator (13). In the mediation model, the mediator is a function of the independent variable and the dependent variable is a function of the mediator. The estimation of each of the mediated effects in the structural equation is used to identify the fact that there is a separation of effects in terms of direct effects and mediated effects (12).

**Figure 3:**
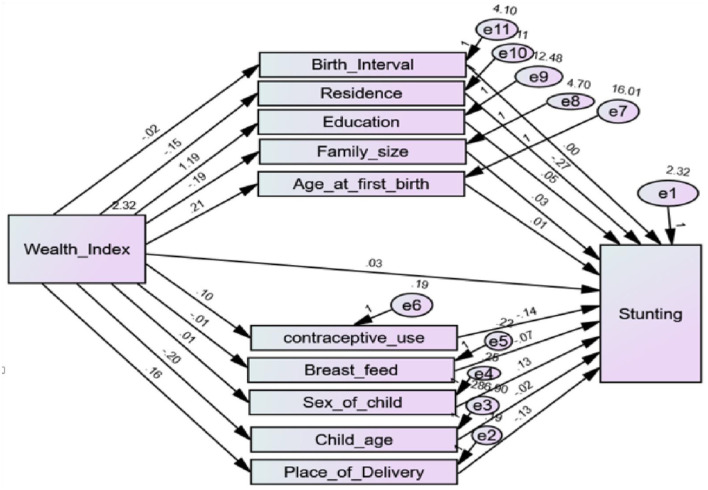
Path diagram of mediators of the link between the wealth index and stunting.

**Figure 4:**
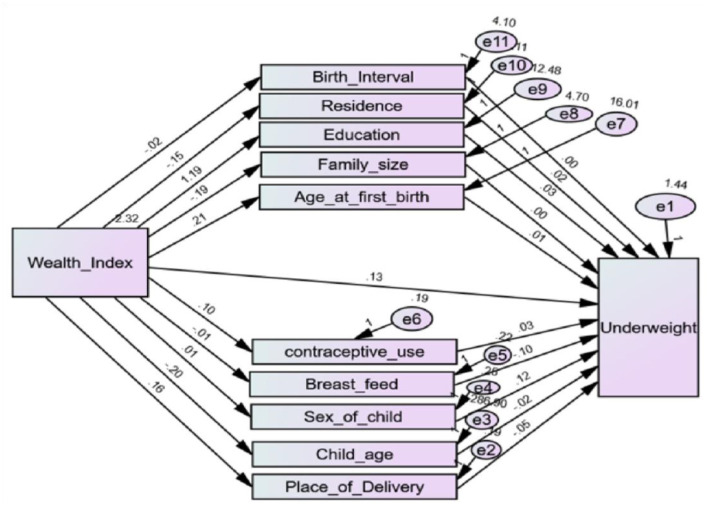
Path diagram of mediators of the link between the wealth index and underweight.

In our case, the direct effect is the pathway from the wealth index to each anthropometric index while controlling for the mediators. Whereas, the indirect effect describes the pathway from the wealth index to each anthropometric indices through the mediator, see Figures 2–4. Finally, the total effect is the sum of the direct and indirect effects of the wealth index on the outcome (15).

### Model fit

All models are open for error but some are better than others. The goodness-of-fit of the model was checked using the normed chi-square (χ^2^) and the comparative fit index (CFI). A normed chi-square statistic value close to 1 and not exceeding 3 indicates a good fit, whereas a CFI statistic value always lies between 0 and 1. A value closer to 1 indicates a very good fit (15).

### Ethics approval

EMDHS program granted permission to download and use the data for this study after being registered and submitting a request with briefly stated objectives of the study. The Institution Review Board approved procedures for DHS public use data sets that do not in any way allow respondents, households, or sample communities to be identified. There are no names of individuals or household addresses in the data files. The detail of the ethical issues has been published in the 2019 EMDHS final report, which can be accessed at: http://www.dhsprogram.com/publications.

The data organization and summary statistics were done using the SPSS 25 software, while other statistical analyses, such as parameter estimation of direct, indirect, and total effects, were done using the AMOS 25 software.

## Results

The frequency distribution and summary statistics of maternal, biological, and environmental characteristics considered in this study are revealed in Table 1. Among 3,918 under-five children involved in this study, 51.4% were male children and 20.2% were from urban areas. The place of delivery of the majority of mothers (56.1%) was at home. The proportion of children from the poorest household (36.4%) was higher as compared to children from poorer (18.3%), middle (14.4%), rich (12.6%), and richest(18.3%) households. Most mothers practiced contraceptive use (68.9%) and refrain from breastfeeding (67.2%). As the children included in this study were aged under 5 years, the maximum age of the child was 59 months. On average each child deviated from the average age of the child (29.54 years) was 16.94 years.

The minimum mother's education level (in years) equals zero indicates the mother was illiterate and on average mother attended education for 2.40 years. The minimum age of the mother at first birth was 12 years, which violates the rule and regulation set by the Ethiopian Ministry of Justice Art.7(1,2), which states that the minimum age of marriage is 18 years. On average, the child's successive birth interval was closer to 3 years. Whereas the minimum birth interval was 1 year, which is too short and may have an adverse effect on the child and maternal health. The anthropometric indicators stunting (HAZ), underweight (WAZ), and wasting (WHZ) were the outcome of interest in the study with standard deviations of 1.58, 1.27, and 1.17, respectively.

**Figure 5:**
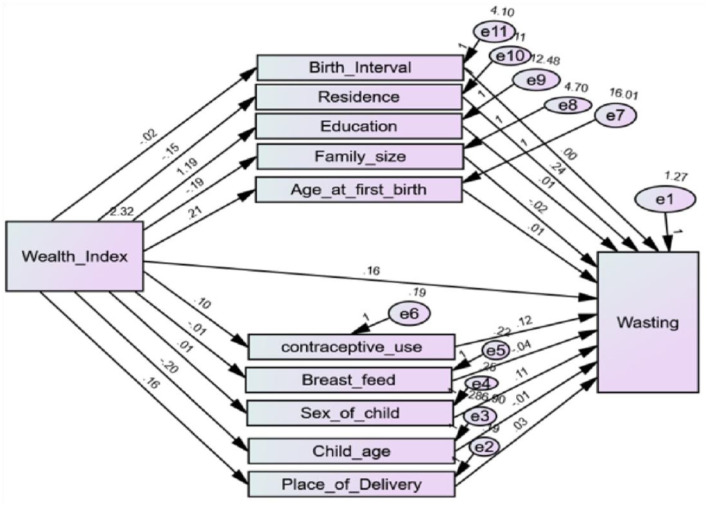
Path diagram of mediators of the link between the wealth index and wasting.

To check the reliability of the hypothesized model in Figure 1, initially, the path diagram that shows direct, indirect, and total effects of wealth index on each anthropometric indicator, such as stunting, underweight, and wasting, was constructed separately in Figures 2–4, respectively. Then, to account for the correlation between anthropometric indices, a path diagram that consists of all anthropometric indices is presented in Figure 5.

The goodness of fit test was checked for each anthropometric index. For instance, the chi-square statistic and CFI for the path model of stunting were 1.290 (*p*-value = 0.685) and 0.952 (closer to 1), respectively, which indicates that the model is not significantly departed from the data. Hence, the model fitted is a good fit for the data. Similarly, the chi-square and CFI statistics of underweight (1.74, 0.557) and wasting (1.021, 0.428) revealed that the model is a good fit for the data.

If a model that is a good fit for the data exists, it does not mean that there are no other models that better fit the data. The goodness of the model is always relative. In the separate model of stunting, underweight, and wasting in Figures 2–4, respectively, the correlation between the anthropometric indices stunting, underweight, and wasting were not taken into account. The chi-square statistic and CFI of the path model in Figure 5 were 0.772 and 0.986, respectively. This indicates that the model improved while the correlation between anthropometric indices is incorporated as compared to the separate one.

The estimated effects of covariates on stunting, underweight, and wasting are depicted in Table 2 and Figures 2–5 as well. The direct effects of these covariates can be obtained simply from the path diagram. Whereas, the indirect and total effects of the wealth index are not directly estimated in the path diagram but in Table 2. The estimated effect of the covariates was estimated using a point and 95% confidence interval. The confidence interval consists of zero, and the *p*-value of <0.05 indicates the corresponding covariates have a significant effect on the corresponding anthropometric indices.

**Table 2:** Parameter estimations of direct, indirect, and total effects of wealth index on stunting, underweight, and wasting.

**Variables**	**Stunting**		**Underweight**		**Wasting**	
	**est. (95% CI)**	***p*-Value**	**est. (95% CI)**	***p*-Value**	**est. (95% CI)**	***p*-Value**
**Direct effects**						
Sex of child: male (*)	0.131 (0.045, 0.200)	**0.016**	0.117 (0.058, 0.177)	**0.012**	0.107 (0.054, 0.173)	**0.005**
Residence: Urban (*)	−0.269 (−0.388, −0.135)	**0.012**	0.023 (−0.057, 0.129)	0.599	0.240 (0.167, 0.332)	**0.009**
Place of delivery: Home (*)	−0.125 (−0.222, −0.034)	**0.014**	−0.054 (−0.122, 0.043)	0.335	0.029 (−0.045, 0.098)	0.483
Contraceptive use: Yes (*)	−0.144 (−0.258, −0.054)	**0.003**	0.026 (−0.042, 0.110)	0.520	0.123 (0.052, 0.178)	**0.015**
Breastfeed: Yes (*)	−0.066 (−0.165, 0.015)	0.182	−0.102 (−0.161, −0.031)	**0.013**	−0.042 (−0.106, 0.029)	0.295
Child age	−0.019 (−0.021, −0.016)	**0.018**	−0.016 (−0.018, −0.014)	**0.019**	−0.006 (−0.008, −0.004)	**0.014**
Birth interval	0.001 (−0.018, 0.019)	0.900	0.000 (−0.017, 0.017)	0.928	0.002 (−0.011, 0.020)	0.702
Mothers age at first birth	0.007 (-0.005, 0.018)	0.287	0.011 (0.003, 0.019)	**0.036**	0.012 (0.003, 0.019)	0.050
Mothers education level	−0.050 (−0.038, −0.060)	**0.014**	−0.033 (−0.024, −0.042)	**0.010**	0.007 (0.000, 0.016)	0.101
Family size	−0.029 (−0.048, −0.011)	**0.013**	0.003 (−0.014, 0.015)	0.846	−0.020 (−0.033, −0.005)	**0.035**
Wealth index	0.026 (−0.012, 0.063)	0.228	0.131 (0.104, 0.164)	**0.006**	0.157 (0.135, 0.187)	**0.005**
**Specific indirect effects**						
Birth interval	0.000 (−0.001, 0.000)	0.743	0.000 (−0.001, 0.001)	0.884	0.000 (−0.001, 0.000)	0.470
Residence: Rural (*)	0.041 (0.021, 0.060)	**0.012**	−0.004 (−0.020, 0.009)	0.599	−0.037 (−0.051, −0.026)	**0.009**
Education	0.059 (0.042, 0.071)	**0.023**	0.039 (0.028, 0.050)	**0.012**	0.009 (0.000, 0.018)	0.112
Family size	−0.005 (−0.009, −0.002)	**0.012**	−0.001 (−0.003, 0.002)	0.768	0.004 (0.001, 0.007)	**0.024**
Mothers age at first birth	0.002 (−0.001, 0.004)	0.196	0.002 (0.001, 0.005)	**0.016**	0.003 (0.001, 0.005)	**0.044**
Contraceptive use: No(*)	−0.014 (−0.025, −0.005)	**0.004**	0.003 (−0.004, 0.011)	0.504	0.012 (0.005, 0.018)	**0.013**
Breastfeed: No(*)	0.000 (0.000, 0.002)	0.214	0.001 (0.000, 0.002)	0.143	0.000 (0.000, 0.002)	0.187
Sex of child: male(*)	0.001 (0.000, 0.003)	**0.014**	0.001 (0.000, 0.003)	**0.019**	0.001 (0.000, 0.003)	**0.009**
Child age	0.004 (−0.001, 0.010)	0.252	0.003 (−0.001, 0.008)	0.252	0.001 (−0.001, 0.003)	0.242
Place of delivery: Home (*)	−0.019 (−0.034, −0.005)	**0.016**	−0.008 (−0.019, 0.007)	0.335	0.004 (−0.007, 0.015)	0.483
**Total indirect effect**	0.069 (0.045, 0.094)	**0.010**	0.036 (0.013, 0.054)	**0.014**	−0.003 (−0.024, 0.012)	0.816
**Total effects**	0.094 (0.063, 0.117)	**0.013**	0.167 (0.147, 0.190)	**0.005**	0.154 (0.135, 0.173)	**0.007**

*, Reference group; OR, odds ratio; CI, confidence interval; est., estimate; SD, standard error.

The bold values indicate the significant variables at 5% level of significance.

Sex of child, residence, place of delivery, contraceptive use, child age, mothers education level, and family size have significant direct effect (*p*-value of <0.05) on stunting of under-five children. The estimated effects of stunting on a female were lower than on male children (1.31:95% CI = 0.450,0.200). As compared to children in rural areas, children in urban areas were less likely to be stunted (0.269; 95%CI = −0.388, −0.135). This indicates the estimated standardized height-for-age (HAZ) of children from rural areas was lower by 0.269 than children from urban areas. The estimated HAZ was increased when the family size decreased (−0.290, 95%CI = −0.048, −0.011). This is in line with the effects of contraceptive use on stunting. Mothers who didn't practice contraceptive use indicate high family size which leads children to expose to malnourished. Children from mothers who practiced contraceptive use were less likely to be stunted as compared to children from mothers who did not practice contraceptive use (−0.144, 95%CI = −0.258, −0.054). Similarly, the sex of the child and child age had a significant effect on both underweight and wasting of under-five children (*p*-value of <0.05). A child who breastfeed had low underweight status as compared to the counterpart (−0.102, 95%CI = −0.161, −0.031). The estimated underweight of a child decreased while the education levels of mothers increased. In contrast, the family size and wasting of the child have a direct relationship, the family size increases as the estimated WHZ of a child become increased. This indicates higher family size contributes to child wasting.

The aim of this study was to determine the potential mediators on the link between wealth and anthropometric indices predominantly, in addition to direct effects, the indirect (*via* mediators) and total effects of the wealth index were also estimated. The indirect effects of the wealth index on stunting through mediators' residence, mothers' education level, family size, contraceptive use, sex of the child, and place of delivery had a significant effect (*p*-value of <0.05). On the other hand, the indirect effects of the wealth index on the underweight through mediators' mothers' education levels, mothers' age at first birth, and sex of child had a significant effect. The estimated total indirect effects of wealth index on stunting and underweight was 0.69 (95% = 0.045, 0.094) and 0.006 (95% = 0.013, 0.054), respectively, and significant (*p*-value of <0.05). Similarly, residence, family size, mothers' age at first birth, and sex of child were the potential mediators of the link between wealth index and wasting. Besides, residence, mothers' education level, family size, contraceptive use, and sex of child were significant mediators of the link between wealth index and stunting. Therefore, residence, mothers' education level, family size, contraceptive use, and sex of child fully mediates the path between wealth index and stunting.

Similarly, mothers' education level, mothers' age at first birth, and sex of child were also significant mediators of the link between wealth index and underweight. The total effects of the household wealth index had a significant effect on stunting, underweight, and wasting. The total effect is an aggregate of indirect and direct effects. The total estimated effects of the wealth index on HAZ, WAZ, and WHZ were 0.094, 0.167, and 0.154, respectively. The positive total estimated effect indicates a direct relationship between the wealth index and the anthropometric indices measures HAZ, WAZ, and WHZ. This indicates higher household income is associated with a lower prevalence of stunting, underweight, and wasting, and vice versa. This shows that the wealth index has a strong contribution to anthropometric indices among children aged 5 years.

The correlations among anthropometric indicators stunting (height-for-age), underweight (weight-for-age), and wasting (weight-for-height) are shown in Table 3. The anthropometric indicator underweights significantly correlated with both stunting (*p*-value = 0.001) and wasting (*p*-value of <0.001), whereas stunting and wasting had a negligible correlation (*p*-value = 0.252). This was also supported in the path diagram consisting of the all pairwise association between anthropometric indicators in Figure 5. Despite insignificant model improvement, for the sake of goodness of fit of the data, the association between stunting and wasting on the path diagram can be removed as the correlation between them was insignificant.

**Table 3:** Covariance and correlation between anthropometric indices.

	**Covariance**	**Correlation**	***p*-Value**
Stunting and underweight	1.27	0.695	<0.001
Stunting and wasting	−0.03	0.017	0.252
Underweight and wasting	0.84	0.622	<0.001

**Figure 6:**
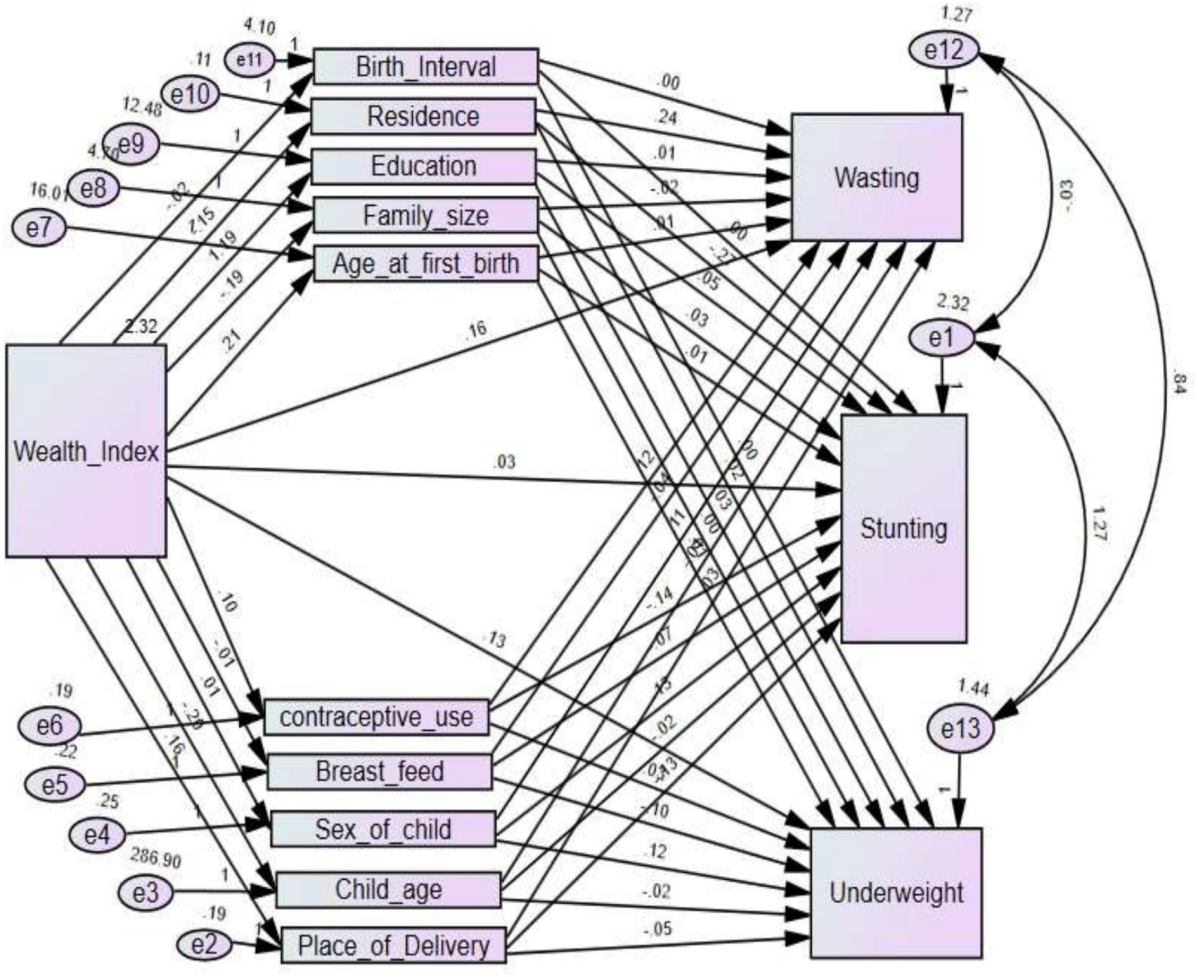
Path diagram of mediators of the link wealth index and the anthropometric indices (underweight, stunting, and wasting).

The statistical significance of the covariance between stunting, underweight, and wasting in Figure 6 can be perceived from Table 3. In Figure 6, the curved two-headed arrow indicates there the association between the two variables. Error terms for a variable are inserted into the path diagram by drawing an arrow from the value of the error term to the variable with which the term is associated.

## Discussion

This study aimed to demonstrate the potential mediators of the link between the household wealth index and the anthropometric indices, such as stunting, underweight, and wasting on under-five children. The mediators include socioeconomic, biological, and behavioral characteristics of the child, mother, and household. The anthropometric indices stunting, underweight, and wasting were computed based on standardized height-for-age (HAZ), weight-for-age (WAZ), and weight-for-height (WHZ), respectively, based on WHO standards. Higher values of HAZ, WAZ, or WHZ indicate lower stunting, underweight, or wasting and vice versa, respectively. According to the WHO standards, a child is stunted, underweight, and wasting if HAZ, WAZ, and WHZ are <-2, respectively. Cross-sectional data obtained from the 2019 Ethiopian Mini Demographic and Health Survey (EMDHS) were the information sources of the analysis. The data management was carried out using SPSS 24, while the parameter estimation of the mediation analysis was done using AMOS 25.

Residence, sex of the child, place of delivery, contraceptive use, mothers' education level, and family size were important determinants of stunting. This was in line with studies in developing countries (3, 8, 10). A majority of mothers (over 55%) are still delivering at home. This leads to a higher likelihood of mother and child mortality during birth. This also shows how the habit of antenatal visits of mothers was insufficient in rural areas in particular. On the other hand, the mother's education level and age at first birth, breastfeeding, child age, and sex had also a significant effect on the underweight. Besides, family size, residence, and contraceptive use were noticed as important determinants of wasting. Likewise, a study by Balogun et al. (2) reported that improving maternal education helps to reduce the venerability of children to malnutrition by implementing effective nutritional methods.

The household wealth index had a significant indirect effect on anthropometric indices through other mediators. In this study, residence, mothers' education level, family size, contraceptive use, and sex of child fully mediates the path between household wealth index and stunting of a child. Similarly, the wealth index had a significant indirect effect on children underweight through mediators on mothers' education level, mothers' age at first birth, and child sex. Whereas, residence, family size, mothers' age at first birth, and contraceptive use significantly mediate the link between household wealth index and wasting. Thus, the household wealth index was an important determinant (significant total effect) of anthropometric indices, such as stunting, underweighting, and wasting likewise of studies in most low and middle-income countries (3, 8, 16).

The low-economic status of the household was associated with stunting, underweight, and wasting. This was consistent with findings in Refs. (8, 10), which reported that children from households with a low wealth index are more likely to be malnourished. Beyond adverse effects on health, anthropometric indices failure exposed children to social insecurity such as school performance (8). The influence of malnutrition on under-five children has worsened which could have a long-run effect on adolescent life (18). In developing countries, such as Africa and Asia and Ethiopia in particular, because of wealth index distraction, numerous households have difficulty in providing food at home for their children and themselves as well (3, 4).

Unlike in a study by Naz et al. (18), in this study, a biological factor such as birth interval was the weakest/null predictor of malnutrition. In Ethiopia, despite the birth interval of most children being short, the likelihood of children to be malnutrition is lower as long as the families have adequate economic status. This is because the household utilizing resources is based on the” less in less out” principle, which indicates that use more if you have more and use little if you have little. Families with enriched economic status are likely to have children with a lower stunting, underweight, and wasting, which is in line with studies in References (3, 10). On the other hand, children from improved socioeconomic status are likely to have children with higher standardized height-for-age (stunting), weight-for-age (underweight), and weight-for-height (wasting) (20).

A study on the determinants of malnutrition among children in rural Kelantan reported that except for environmental factors, biological and behavioral factors do not have a substantial link with children's malnutrition (16). In contrast, in this study beyond environmental factors, biological and behavioral factors, such as family size, mothers' age at first birth, residence, and contraceptive use, were also potential determinants linked with children's malnutrition. Therefore, the government and other stakeholders need to give higher attention to empowering the households' monetary dimension to reduce the prevalence of malnutrition in children by considering potential mediators of the link between economic status and malnutrition.

## Conclusion

Residence, mothers' education level, family size, contraceptive use, and sex of child were potential mediators of the link between wealth index and stunting. Whereas, mothers' education level, mothers' age at first birth, and sex of child were also significant mediators of the link between the wealth index and underweight. Besides, residence, family size, mothers' age at first birth, and sex of child fully mediate the path between the wealth index and wasting. This indicates environmental, maternal, biological, and behavioral factors are the potential mediators of the link between the wealth index and anthropometric indices. As a result, the mediation analysis of the link between the wealth index and the anthropometric indices enables policymakers and planners at the personal and governmental levels to identify more efficient, alternative intervention strategies for malnutrition in under-five children.

This study is not without limitations. It is conducted based on cross-sectional data and hence not assessed based on the prevalence of undernutrition and its direct or indirect factors over time. Besides, several additional socioeconomic, demographic, biological, and behavioral characteristics were not considered. Thus, we would like to recommend that future researchers considered these characteristics as they might affect undernutrition directly or indirectly.

## Data availability statement

The data that the authors used to produce this manuscript are available upon reasonable request from demographic and health survey (DHS) cite www.dhsprogram.com. The DHS Program is authorized to distribute, at no cost, unrestricted survey data files for legitimate academic research. Registration is required for access to data.

## Ethics statement

The studies involving human participants were reviewed and approved by the ethical clearance for 2019 EMDHS was approved by the Ethical Review Board of Ethiopia Central Statistical Agency (ECSA). Since the study was a secondary data analysis of publicly available survey data from the MEASURE DHS program, ethical approval and participant consent were not necessary for this particular study. We requested DHS Program and permission was granted to download and use the data for this study from www.dhsprogram.com. We confirm that all methods were carried out following the relevant guidelines and regulations. Written informed consent for participation was not required for this study in accordance with the national legislation and the institutional requirements.

## Author contributions

LT had the idea for the study, proposed the first draft, conducted data analysis, and interpretation, and wrote the manuscript. AW edited and revised the manuscript. All authors read and approved the final manuscript.

## Conflict of interest

The authors declare that the research was conducted in the absence of any commercial or financial relationships that could be construed as a potential conflict of interest.

## Publisher's note

All claims expressed in this article are solely those of the authors and do not necessarily represent those of their affiliated organizations, or those of the publisher, the editors and the reviewers. Any product that may be evaluated in this article, or claim that may be made by its manufacturer, is not guaranteed or endorsed by the publisher.

## Abbreviations

EMDHS: Ethiopian mini demographic and health survey
CI: Confidence interval
SEM: Structural Equation Modeling
SDGs: Sustainable Development Goals
CIAF: Composite Indices of Anthropometric Failure
CFI: Composite Fit Index
WHO: World health organization.
